# Supplementary material for: Exploring the role of stromal osmoregulation in cancer and disease using executable modelling
Source: Nat Commun. 2018 Aug 1;9:3011. doi: 10.1038/s41467-018-05414-y (PMC6070494; doi:10.1038/s41467-018-05414-y)
Supplement: Supplementary file 1 — Supplementary Information [file 41467_2018_5414_MOESM1_ESM.pdf]

Supplementary Information

EXPLORING THE ROLE OF STROMAL OSMOREGULATION IN CELLULAR TRANSFORMATION IN CANCER AND DISEASE USING EXECUTABLE MODELLING

David Shorthouse, Angela Riedel, Emma Kerr, Luisa Pedro, Dóra Bihary, Shamith Samarajiwa, Carla Martins, Jacqueline Shields, Benjamin A Hall

Supplementary Note 1- Choice of modelling approach

In this study we elected to use an executable modelling approach to explore the role of osmoregulation in the cell (Available publicly at http://biomodelanalyzer.org). Here, protein and gene networks are represented at a high level of abstraction, in order to reproduce the behaviour at a functional level rather than in terms of the precise chemistry or physics. Compared with conventional, continuous approaches there exist several specific advantages that make this particular paradigm appropriate. In the approach used in this paper, the underlying variables within the model are discrete and each have a finite set of states. Transitions between states are determined according "target-functions" that effectively interpret how the variable responds to dependencies similar to a truth-table.

This technique allows modelling in the absence of complete knowledge of all interacting entities. For example if we know upregulation of a certain gene product impacts the behaviour of an expressed protein within a system, we can include that interaction even if we do not have information on the intermediaries. Additionally, the executable approach allows the generation of useful and interpretable networks in the absence of precise kinetic data, which is absent for many membrane transport and ion channels and their regulators.

A further advantage in using executable models is the ability to use proof based approaches ("model checking") to analyse the system. Whilst it is possible to run simulations of the system, the proof based approach takes advantage of bespoke algorithms and symbolic representations offer guarantees of model behaviour under all circumstances. Stability analysis for example, addresses all terminal states of a model (where a stable model is said to have a global, fixpoint attractor). Stability analysis will therefore return a result stating that a given model is either stable, bifurcates, or ends in a cycle, from all initial states. The particular algorithm used for stability analysis in the BMA is described in Cook et al[1] . In this algorithm the proof of stability is achieved by reasoning over the behavior of individual variables repeatedly until either all variable ranges can be reduced to a single value or all possible approaches are exhausted. A SAT solver is used to symbolically prove the stability or find counter examples if stability has not been proved in the first step, using the reduced ranges already calculated. Other analyses are also available, with one example being specifications encoded in linear temporal logic (LTL) [2]. In both stability testing and LTL model checking, these analyses return results that describe the behaviour of the model across all state space, and so can guarantee a behaviour does or does not exist.

This approach cannot generate precise, quantitative outputs, due to the choice in using discrete states. For example, this may mean that you are unable to ascertain specific concentration changes of any member of the network, only that the concentration is likely to

increase or decrease. As such, the broad thresholds used in the discretization process may mask smaller, but statistically significant changes in activity or concentration.

Model quality is assessed by comparing behaviours of the model with a “formal specification”. This specification lists the specific properties expected of the model based on input data. For example, this may include a requirement the model is stable and a set of variables have specific assignments at the fixpoint. If the model violates any part of this specification it is not considered correct and requires refinement. Features that are not included in this specification may be used for prediction. Typically a model may include cellular phenotypes, morphology, or other measurable changes, and these can be both used validate that the model is correct, and assess the accuracy depending on what is kept in the specification. Systems built with executable models are generally coarse (use a small range of variables), because this keeps the systems tractable for model checking and other proof techniques. In this study we used outputs with a range of 5 (0 – significantly decreased; 1 – decreased; 2 – unchanged; 3 – increased; 4 – significantly increased), though generally experimentally and from literature searches it is hard to quantify “significantly” versus a normal increase, as such we treat both similarly. All models described in the text are available at http://dx.doi.org/10.5281/zenodo.1257326.

Supplementary Note 2 - Proliferation vs Viability

There is complexity in the literature surrounding the exact definitions of proliferation and viability, with the terms often being used interchangeably or potentially incorrectly. Metabolic assays for example are generally cited as measuring viability, but have been explicitly linked to proliferation[3]. Whilst there is a relationship between viability and proliferation (in particular, an increase in viability implies an increase in proliferation or proliferative potential), this relationship is not necessarily linear. For example, a decrease in cellular viability does not necessitate a similar decrease in proliferation, and cells that proliferate to a higher degree than a control set, but also die more, may have comparable viability. Due to the complexity of the relationship, we have chosen to include viability in our model, rather than proliferation. Viability serves as a better description of the behaviour captured, and should reflect the behaviour observed in most experiments more accurately than proliferation. We note that we have performed both proliferation and viability assays for siRNA knockdown experiments, and note that in the case of SLC9A1, the assays give different results.

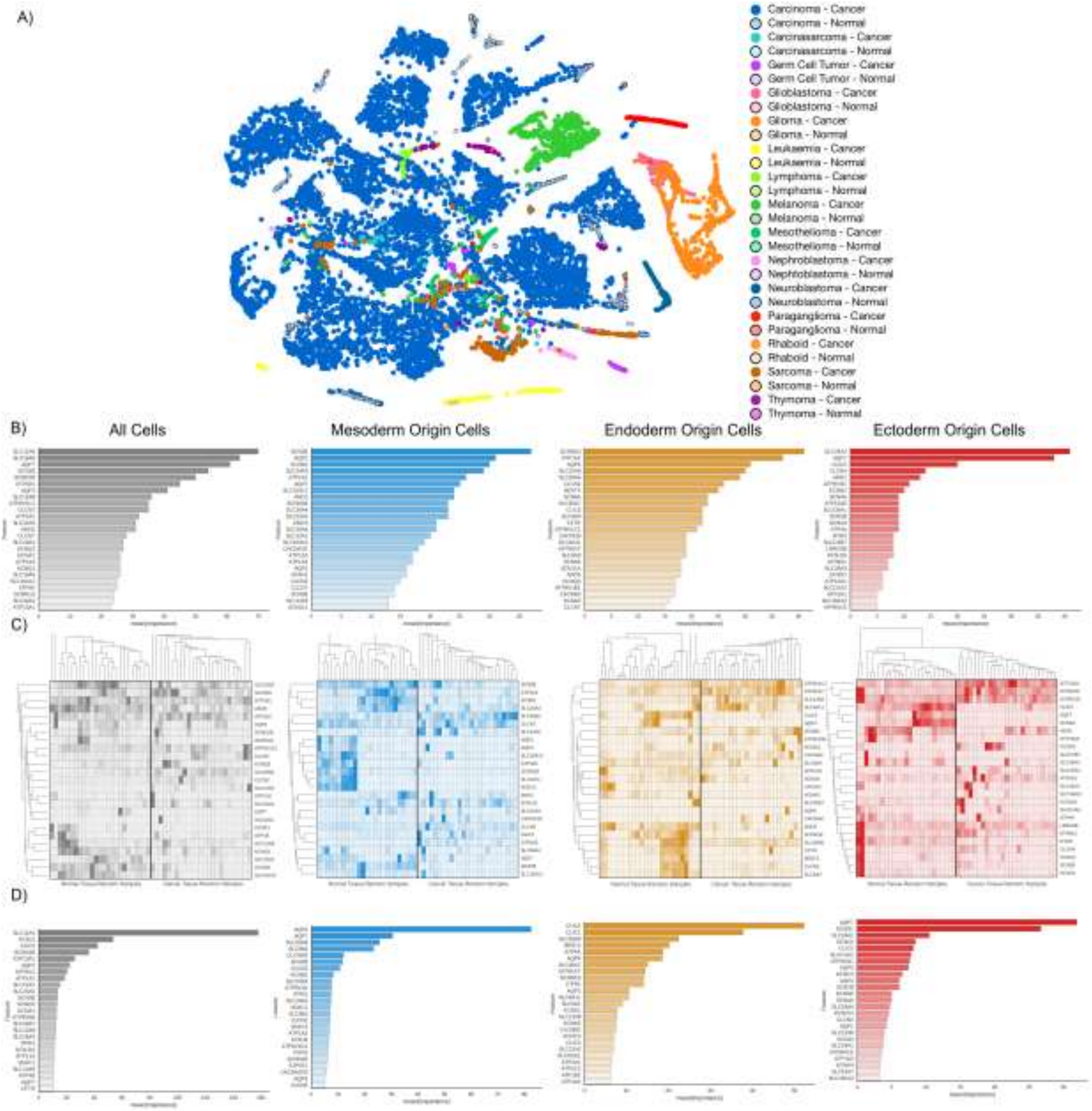

Supplementary Figure 1: A) t-SNE plot of the cancer landscape when expression of 380 membrane transporters is used. Each point represents a single patient sample from the TCGA database, and is coloured by it’s tissue of origin. B) Feature weights for different subsets of data based on cellular embryonic origin. Feature weights represent genes most used to distinguish between binary classes within the predictive model, shown are all samples (grey), samples on from mesoderm embryonic origin (blue), samples from endoderm origin (orange), and samples from ectoderm origin (red). C) Heatmaps of random subsets of 25 non-cancer, and 25 cancer samples, showing the expression levels of the top weighted 25 genes. D) Feature gain rankings for cells of different embryonic origins, feature gain represents the relative degree to which that specific feature influences the decision of the algorithm to label a cell cancer or non-cancer.

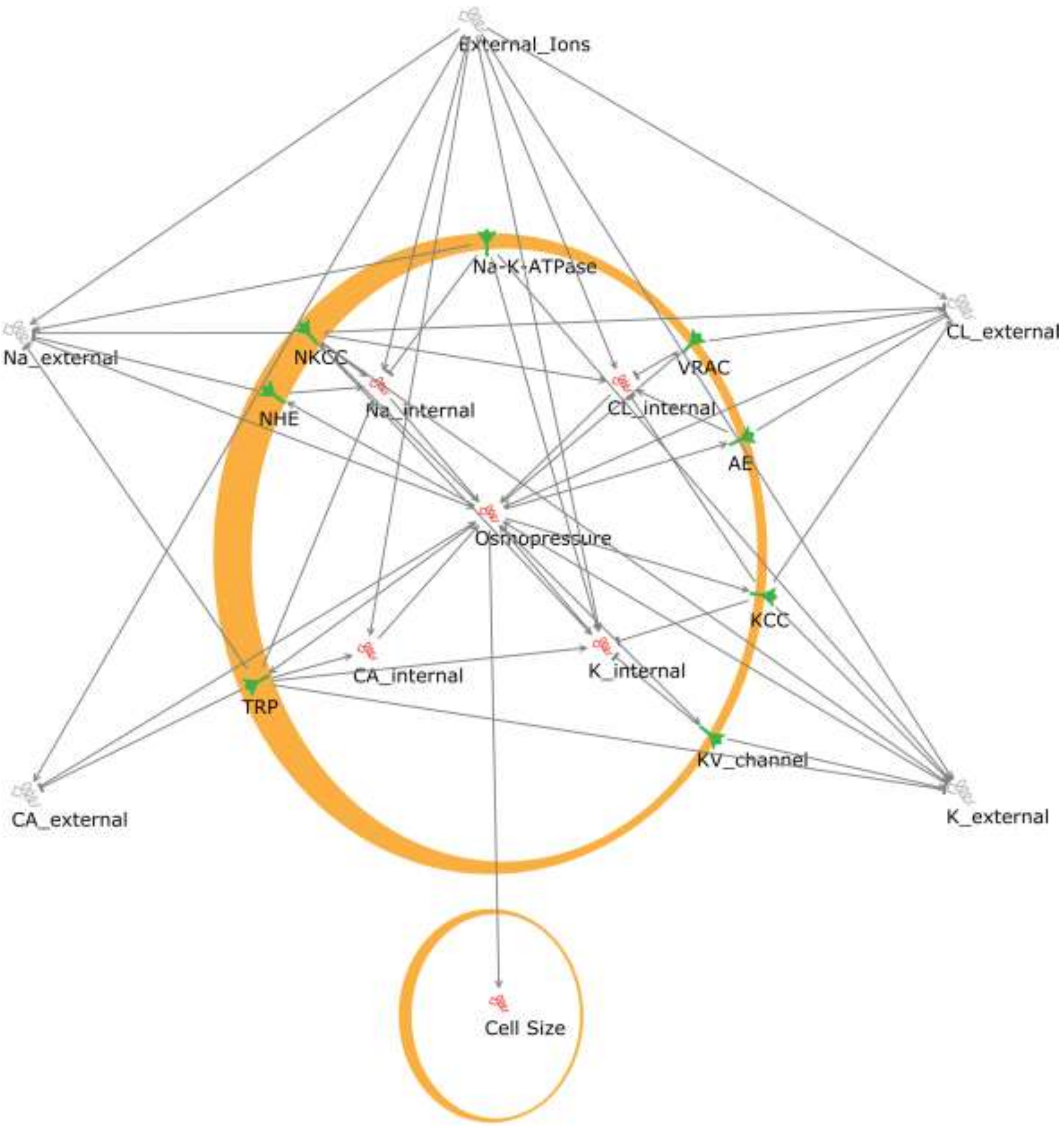

Supplementary Figure 2: Network model of canonical response to osmoregulation shown in the Biomodelanalyzer interface. Includes are abstract nodes for the concentration of external ions, the osmotic pressure felt by the cell, and the cell size. The model responds to changes in osmotic pressure that the cell feels by upregulating transport proteins that shuttle ions to rectify any differences across the membrane. The total model includes 19 nodes.

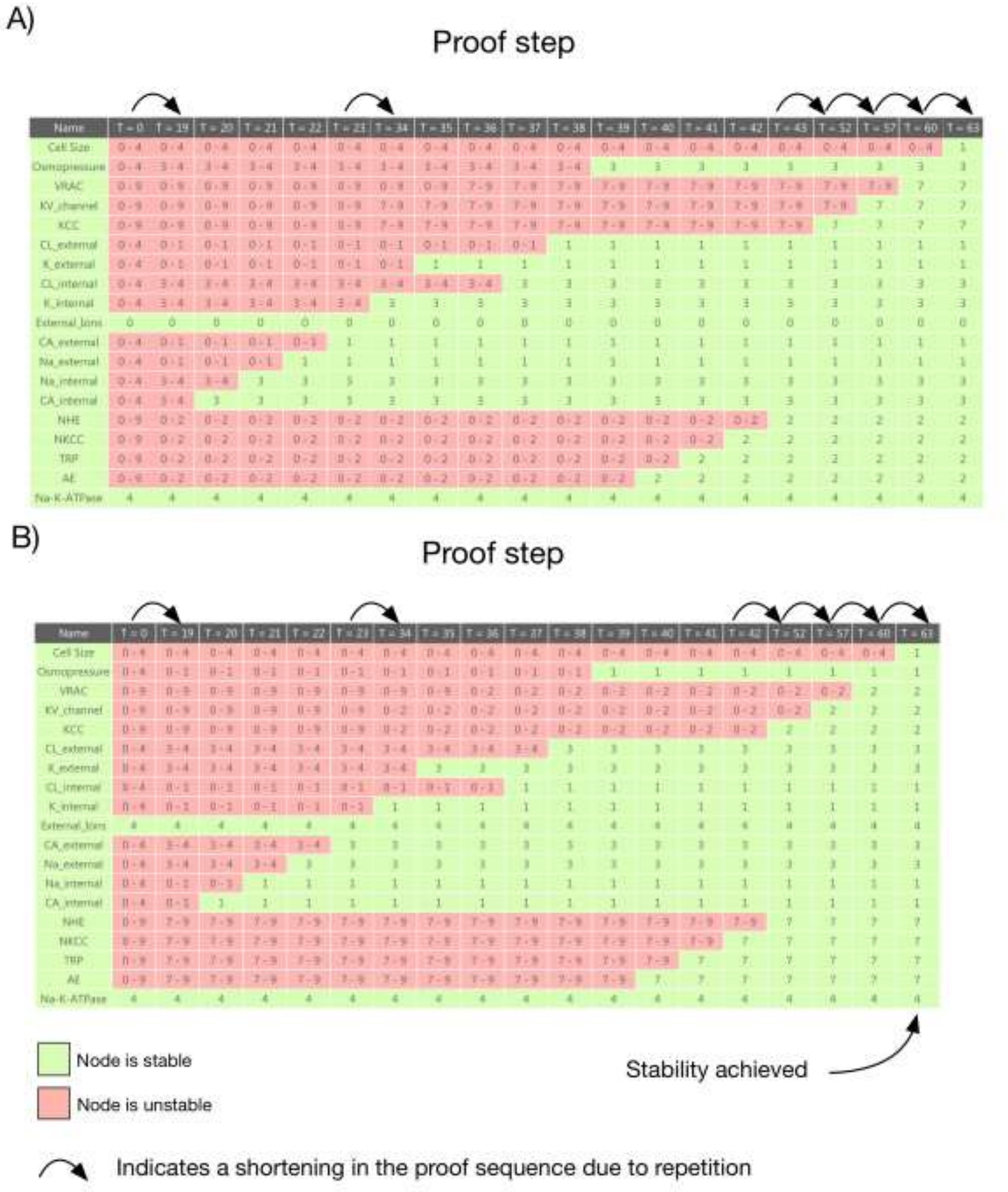

Supplementary Figure 3: Proof progression for the model of canonical osmoregulation under hypertonic conditions. The proof progression is shown in the biomodelanalzyer interface when stability analysis is run, and a proof indicates that all simulations of the system within current bounds will end in the same self-renewing state. Each cell represents the value range of a particular node at a particular stage of the proof progression. A cell coloured green has stabilized to a single value, a cell coloured red has a range of values that can occur at that stage of the proof. The proof ends with all cells within the model returning a stable value, indicated by the green cells at the bottom of the plot. Proofs are shown for models in A) Hypertonic stress, and B) Hypotonic stress.

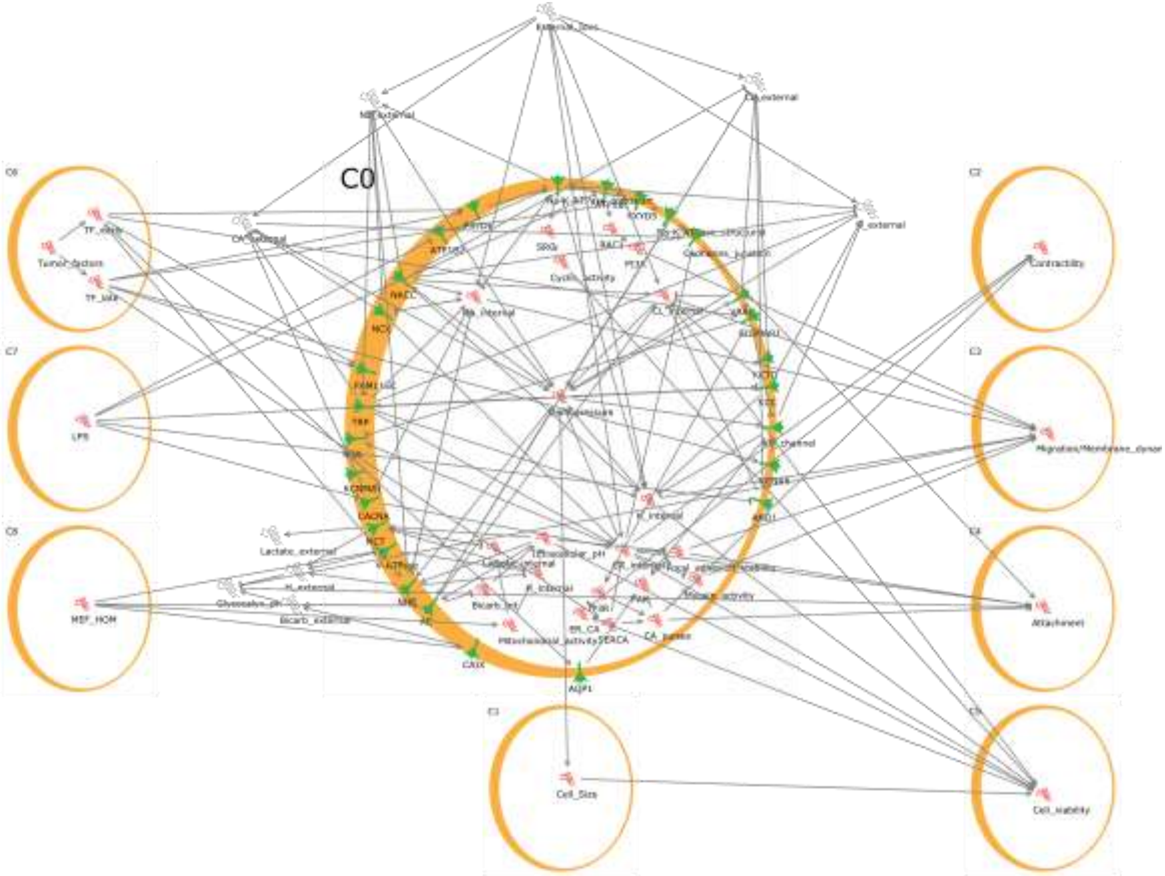

<u>Supplementary Figure 4:</u> Full model of ionic transport in a mammalian cell, shown in the BioModelAnalyzer interface. Included are nodes representing phenotype outputs (right), of cell size, cell contractility, cellular movement/migration, cellular adhesion, and cellular proliferation. The model also contains nodes referring to specific experimental conditions (left), which set protein and gene activity levels to those within experiments. Upon activation of a condition, there is a concerted change in the expression of many channels influenced by movement of ions and protein activation cascades, before an emergent cellular phenotype is observed through the phenotype nodes.

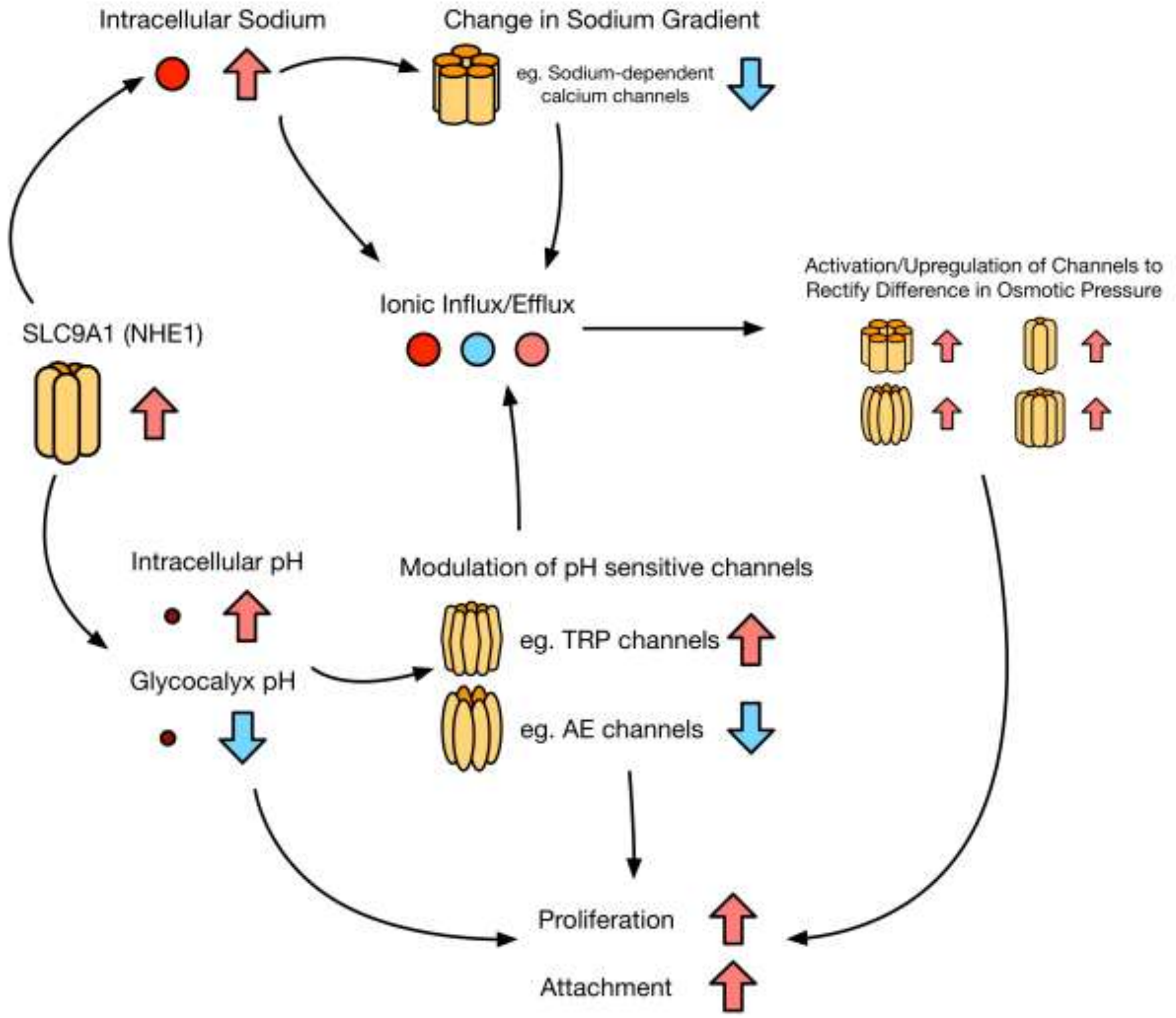

<u>Supplementary Figure 5:</u> Schematic of the signalling cascade occurring in the model when the SLC9A1 (NHE1) node has its value increased. The increase in SLC9A1 results in an increase in extracellular sodium, and a concurrent increase in intracellular pH and reduction in the pH of the Glycocalyx. These lead to changes in the activation state of other channels such as sodium dependent calcium channels, which lead to larger emergent changes in ionic concentrations. These combined behaviours eventually result in an increase in proliferation and attachment

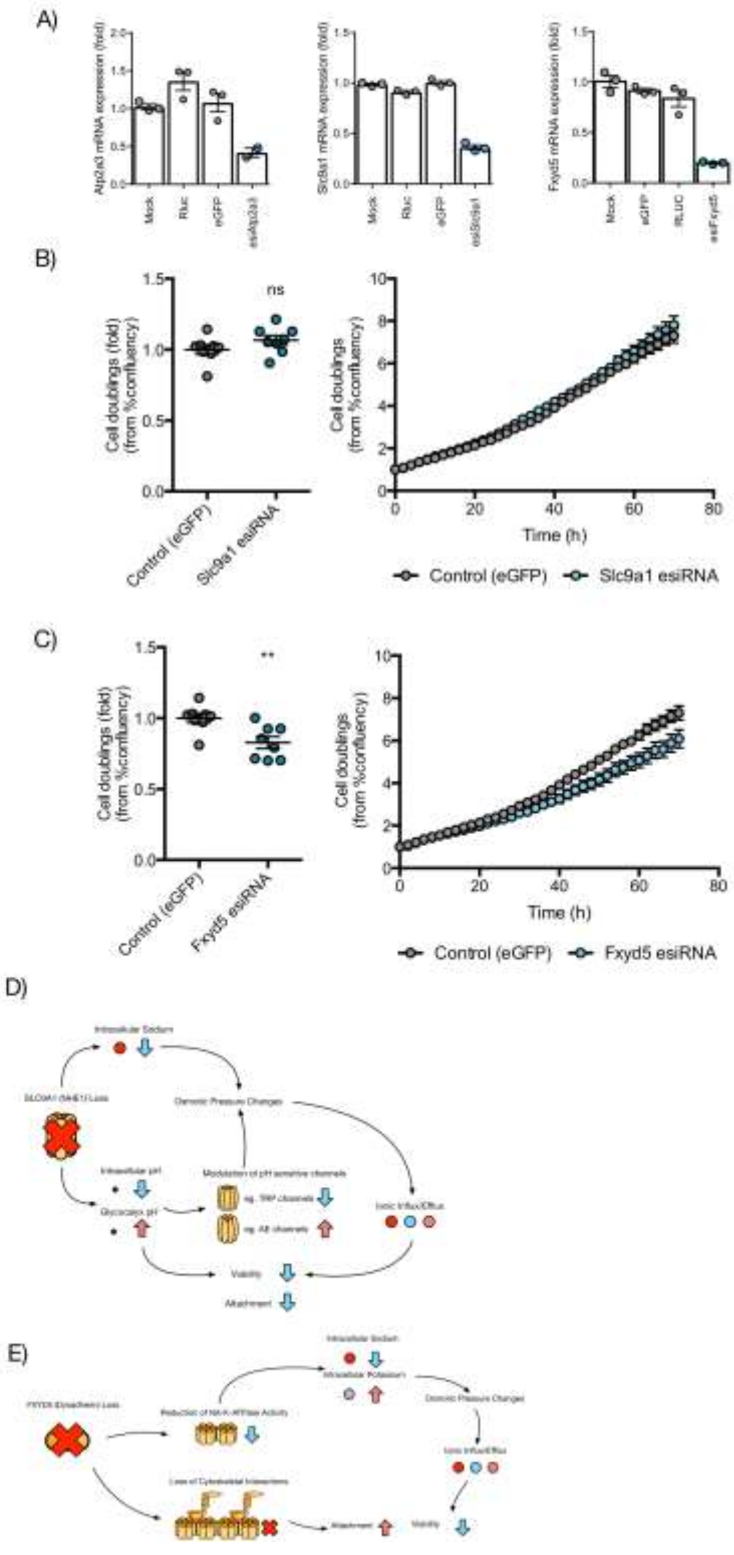

Supplementary Figure 6: A) qPCR validation of siRNA knockdowns of ATP2A3, SLC9A1, and FXYD5 in tumor factor exposed FRCs. B) Proliferation plot for FRCs after siRNA knockdown of SLC9A1. C) Proliferation plot for FRCs after siRNA knockdown of FXYD5. D) Knockdown cascade predicted by the model upon loss of SLC9A1 (NHE1). E) Knockdown cascade predicted by the model upon loss of FXYD5 (Dysadherin)

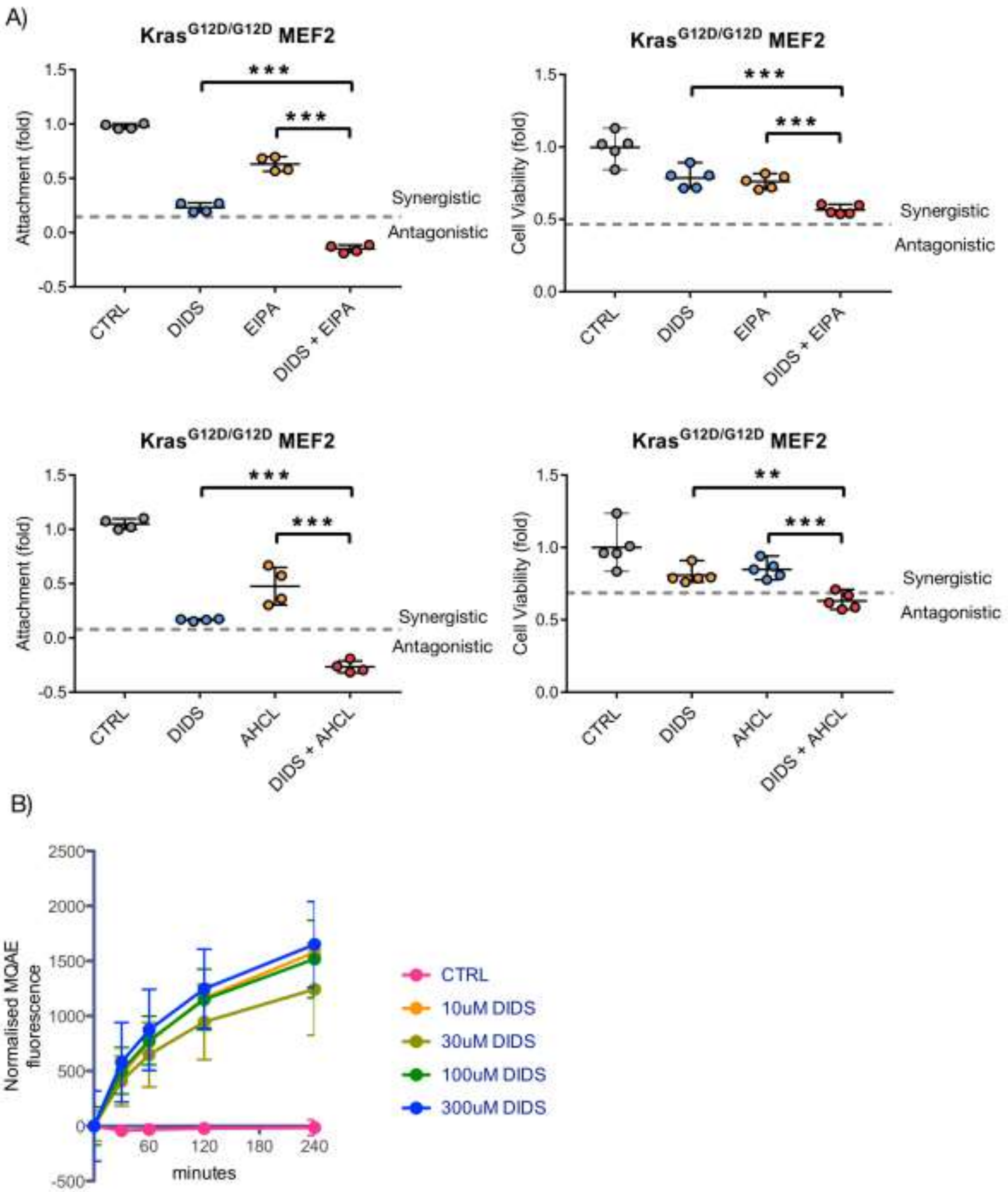

Supplementary Figure 7: A) biological repeats of the application of 10uM DIDs ± 10nM EIPA or AHCL in Kras$^{G12D/G12D}$ MEFs. Shown are the effects on: Attachment with EIPA, Viability with EIPA, Attachment with AHCL, Attachment with AHCL. *P <0.05, **P <0.01, ***P<0.001 with two tailed unpaired t-test.) Loss of intracellular chloride measured by MQAE fluorescence, indicating that addition of DIDS blocks AE chloride channels in MEFs, and therefore lowers intracellular chloride. Dotted line indicates Bliss Independence value.

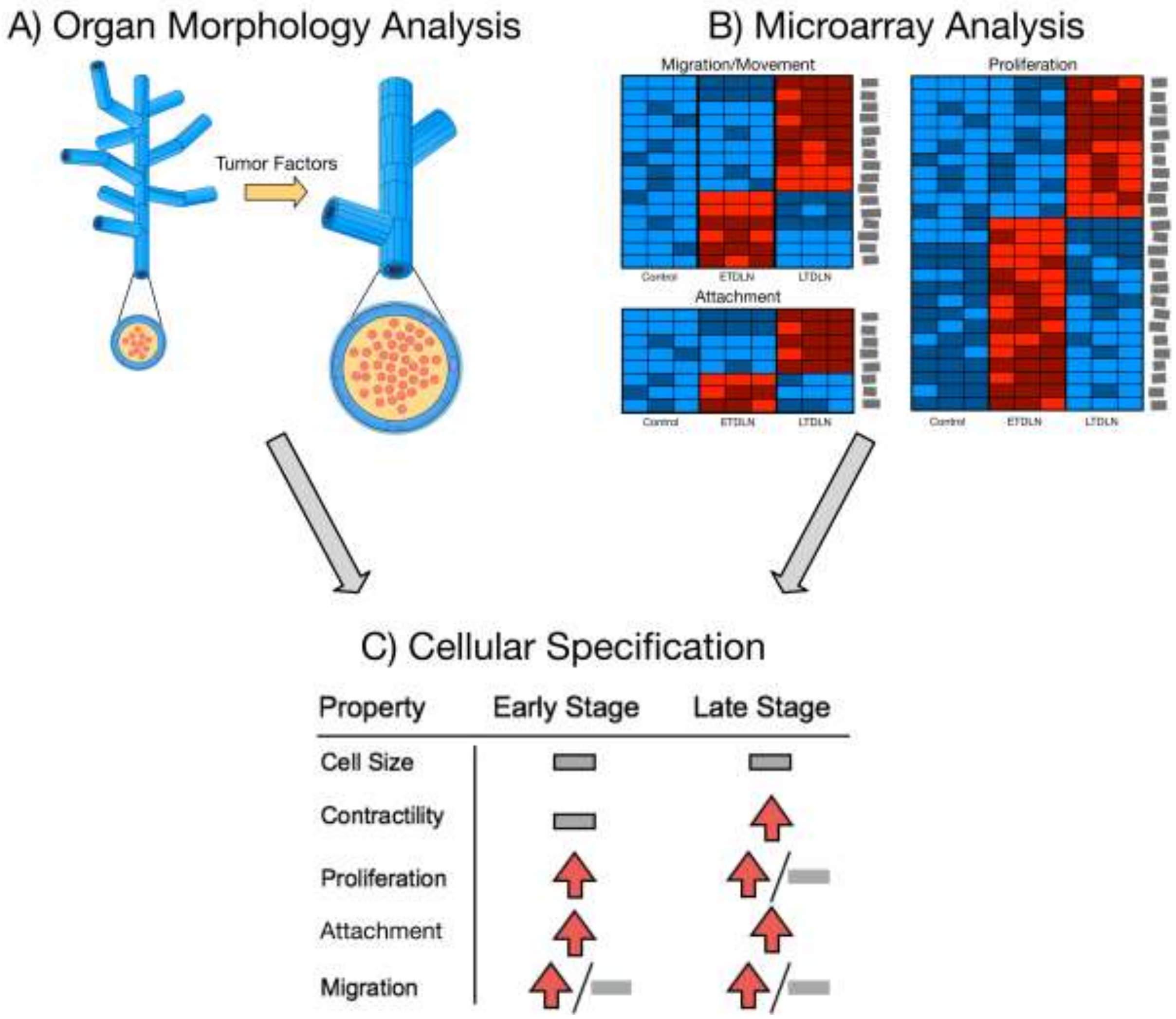

Supplementary Figure 8: Generation of a specification for cellular behaviour based on experimental data. A) Organ or tissue level morphology data in the LN is used to imply behaviours of single cells. A large organ-level restructuring indicates cells are proliferating (as the organ swells with cells), and undergoing membrane changes/movement. Organs are also more resistant to stretching and rigid, and thus indicate and increase in contractility. Additionally, light scattering studies indicate that individual cells do not change size. B) Microarray analysis from LN FRCs highlights key deregulated pathways of genes involved in specific cellular behaviours, such as attachment, migration, and proliferation. By combining these two sources of information on cellular behaviour we generated a cellular specification C). Cellular specification describing the phenotype of individual FRCs in terms of size, contractility, proliferation, attachment, and membrane dynamics/movement. This specification is used as a target for generation of an initial model of transport proteins and phenotype

Supplementary References:

1. Cook, B. *et al. Computer Aided Verification*. *Lecture Notes in Computer Science (including subseries Lecture Notes in Artificial Intelligence and Lecture Notes in Bioinformatics)* **8559,** (2014).

2. Claessen, K., Fisher, J., Ishtiaq, S., Piterman, N. & Wang, Q. Model-Checking Signal Transduction Networks through Decreasing Reachability Sets. (2011)

3. Yang, D. Reduced expression of FXYD domain containing ion transport regulator 5 in association with hypertension. *Int. J. Mol. Med.* (2011).
