# Supplementary material for: Exploring the role of stromal osmoregulation in cancer and disease using executable modelling
Source: Nat Commun. 2018 Aug 1;9:3011. doi: 10.1038/s41467-018-05414-y (PMC6070494; doi:10.1038/s41467-018-05414-y)
Supplement: Supplementary file 3 — Description of Additional Supplementary Files [file 41467_2018_5414_MOESM3_ESM.pdf]

Description of Additional Supplementary Files

File Name: Supplementary Data 1
Description: List of ion channel genes used for binary classification.

File Name: Supplementary Data 2
Description: List of random genes used for binary classification excluding ion channels.

File Name: Supplementary Data 3
Description: Specification for cellular behaviours related to ionic concentrations and membrane proteins. Included is how the property influences a cellular behaviour, and how altering the nodes will influence cellular phenotype in the model.

File Name: Supplementary Data 4
Description: List of genes chosen for further study using the model, included is the gene name, and references related to their impact on cellular morphology, particularly focussing on cancer.

File Name: Supplementary Data 5
Description: Justification for biological effect of nodes in the model, included are references for specific behaviour changes and interactions.

File Name: Supplementary Data 6
Description: All models used in the manuscript.
